# Perioperative suprachoroidal hemorrhage and its surgical management: a systematic review

**DOI:** 10.1186/s40942-024-00577-x

**Published:** 2024-08-21

**Authors:** Margarida Ribeiro, David Matos Monteiro, Ana Filipa Moleiro, Amândio Rocha-Sousa

**Affiliations:** 1 Department of Ophthalmology, Unidade Local de Saúde de São João, Alameda Prof. Hernâni Monteiro, Porto, 4200-319 Portugal; 2 Department of Biomedicine, Unit of Pharmacology and Therapeutics, Faculty of Medicine, University of Porto, Porto, Portugal; 3 Faculty of Medicine, University of Porto, Porto, Portugal; 4 Department of Surgery and Physiology, Faculty of Medicine, University of Porto, Porto, Portugal

**Keywords:** Perioperative period, Choroidal detachment, Choroidal hemorrhage, Drainage, Vitreoretinal surgery, Visual acuity

## Abstract

**Purpose:**

Suprachoroidal hemorrhage (SCH) is a rare but severely feared sight-threatening complication of intraocular surgery, and its management remains debatable. We intended to summarize the existing surgical management options regarding perioperative SCH, describing different techniques and their respective visual outcomes.

**Methods:**

A systematic literature search of articles published since 1st January 2011 until 31st December 2022 was performed using MEDLINE (PubMed) and Scopus. Eligibility criteria included the adult population with SCH related to intraocular surgery.

**Results:**

Thirty-eight studies enrolling 393 patients/eyes were assessed after a selection process among 525 records. We included 5 retrospective cohort studies, 15 case series and 18 case reports. We documented cases of acute SCH diagnosed intraoperatively and delayed SCH, treated until a maximum of 120 days after the diagnosis. Best corrected visual acuity at diagnosis was generally poor, with variable final visual outcomes. Techniques of external drainage with or without combined pars plana vitrectomy (PPV), type of endotamponade (if PPV performed), anterior chamber maintainer and reports of the use of recombinant tissue plasminogen activator were described.

**Conclusion:**

To the best of our knowledge, this is the first systematic review assessing perioperative SCH and its surgical management. There is no standardized surgical approach of SCH and longitudinal intervention studies are lacking. To ensure that patients achieve the best possible visual outcome, prompt diagnosis and treatment are crucial. Therefore, further clinical research is on demand to improve the management of this clinical sight-threatening entity.

## Key messages

• Suprachoroidal hemorrhage is a rare but severe sight-threatening complication of ocular surgery.

• Longitudinal intervention studies regarding its surgical approach are very scarce.

• This review summarizes the recent evidence on several surgical techniques and their respective visual outcomes.

## Introduction

Suprachoroidal hemorrhage (SCH) is a very rare but severely feared sight-threatening condition that can occur related to trauma or as a complication of intraocular surgery [1, 2].

The suprachoroidal space is considered a potential space between the sclera and the choroid, which allows the drainage of the uveoscleral pathway. It contains the long and short posterior ciliary arteries and when blood from these arteries accumulates in this space, a SCH is formed [3].

The onset of SCH is thought to be related with a sudden decrease in intraocular pressure (IOP), which can cause fluid diffusion from the vortex veins into the suprachoroidal space, creating a suprachoroidal effusion. This can cause stretching of the posterior ciliary arteries, leading to their rupture, thus forming a SCH [4].

SCH can be classified according to its etiology (spontaneous, perioperative or trauma-related), size or extent of hemorrhage (number of quadrants involved), degree of elevation (including kissing choroidals) or its relation with ocular surgery (intraoperative/expulsive/acute or postoperative/delayed) [5].

Perioperative SCH are the most common. Its incidence varies according to the type of surgery. SCH occurs less commonly in association with modern techniques of cataract surgery, having an estimated incidence between 0.03 and 0.1% [6], although in the past it could happen in up to 0.81% of these surgeries [7], due to older techniques such as extracapsular cataract extraction (ECCE) in comparison to phacoemulsification [3, 5].

Unlike cataract surgeries, where the majority of SCH tend to occur intraoperatively, in glaucoma surgeries it is more frequently a postoperative/delayed complication [3, 8], with higher incidence rates that range from 0.15 to 6.1% [9, 10]. In vitreoretinal surgery, it varies from 0.17 to 1.9% [11, 12] and in penetrating keratoplasty (PK) from 0.45 to 1.08% [13, 14].

There are different systemic and ocular risk factors that increase the likelihood of perioperative SCH. The systemic risk factors include advanced age, atherosclerosis, uncontrolled hypertension, diabetes, and use of anticoagulants and antiplatelet agents. Ocular risk factors encompass choroidal arteriolar sclerosis, glaucoma, high myopia, aphakia or pseudophakia, previous intraocular surgery or trauma, and increased IOP or SCH in the other eye [8, 15, 16].

It is also possible to distinguish between intraoperative and postoperative risk factors. The former include a sudden decrease in IOP, retrobulbar and general anesthesia, intraoperative hypertension, Valsalva maneuver, posterior capsular rupture with vitreous loss, ECCE and prolonged surgical time, while the latter include ocular hypotony, trauma and Valsalva maneuver, as observed when coughing or vomiting [3, 8, 17].

SCH may be diagnosed clinically. Intraoperatively, SCH can present with a shallow anterior chamber (AC) with mild cell and flare, loss of the red reflex, high IOP (subjectively noticed by hardening of the eye), posterior capsule bulging and extrusion of intraocular contents (lens, iris, retina and vitreous prolapse). In the postoperative period, SCH can be accompanied by sudden onset of excruciating eye pain (frequently 1–5 days after surgery), headache, nausea, vomiting, decreased visual acuity (VA) and the aforementioned signs also present in the intraoperative period [8, 18–20].

During the evaluation of SCH, ultrasound can be an important tool to characterize several features, to help rule out other pathologies, to guide treatment and monitor its progress. This imaging technique can be essential to assess retinal involvement, vitreous hemorrhage, the type of SCH, its size/extent, evolution and the best timing for surgical drainage, if needed [5, 8].

When a SCH is noted intraoperatively, in order to avoid extrusion of intraocular contents (expulsive SCH), the surgery should be promptly finished, with rapid suture closure and eventually creating immediate posterior draining sclerotomies, which remains questionable. Postoperatively, general treatment can include topical or oral steroids and cycloplegic agents like atropine in order to reduce inflammation and pain, as well as oral analgesics (except aspirin and non-steroidal agents, which are contraindicated). IOP-lowering drugs such as topical beta-blockers or oral carbonic anhydrase inhibitors are also used [3, 8, 18].

Since SCH is a very uncommon complication, there are no randomized controlled trials and longitudinal studies regarding its surgical approach are very scarce. Most of the evidence found in the literature is limited to case series and case reports. There is a lot of controversy and lack of agreement concerning the decision for surgery, the optimal timing for drainage of the SCH and the surgical approach itself [8, 21].

Therefore, this systematic review intends to revisit the state-of-the-art of the surgical management of perioperative SCH, describing several surgical techniques and respective visual outcomes regarding this dreaded complication.

## Methods

### Eligibility and search strategy

This systematic review was conducted according to the PRISMA (Preferred Reporting Items for Systematic Reviews and Meta-Analyses) statement [22].

A systematic literature search of articles published since 1st January 2011 until 31st December 2022 was performed on January 6th, 2023, in two electronic databases: MEDLINE (PubMed) and Scopus. Full details of the search regarding the query used are presented in Table 1. Eligibility criteria included the adult population with SCH related to intraocular surgery (intraoperative/ expulsive/acute or postoperative/delayed). Exclusion criteria included reviews, studies with no available full-text, articles not conducted in humans, videos, comments and articles not including surgical therapeutic options.

**Table 1:** Keywords used to perform the query in the two databases used in this study (date of search = 6th of January 2023)

Scopus	(TITLE-ABS-KEY(“choroid hemorrhage”) OR TITLE-ABS-KEY(“hemorrhagic choroidal detachment”) OR TITLE-ABS-KEY(“suprachoroidal hemorrhage”)) AND (TITLE-ABS-KEY(vitrectomy) OR TITLE-ABS-KEY(surgery) OR TITLE-ABS-KEY(sclerotomy))
MEDLINE / PubMed	(“Choroid Hemorrhage*“[MeSH Terms] OR “Choroid Hemorrhage*“[Title/Abstract] OR “Hemorrhagic choroidal detachment*“[Title/Abstract] OR “Suprachoroidal hemorrhage*“[Title/Abstract]) AND (“Vitrectomy“[MeSH Terms] OR “Vitrectomy“[Title/Abstract] OR “Surgery“[Title/Abstract] OR “Sclerotomy“[Title/Abstract])

### Study selection, data collection process, study outcomes and quality assessment

Two independent investigators (M.R. and A.F.M.) screened the title and abstract of all the studies from the initial search and selected potentially relevant studies. The full-text was retrieved for studies meeting the eligibility criteria or for doubtful eligibility from the title and abstract. Two reviewers (M.R. and D.M.) independently assessed the full-text for inclusion. Discordant decisions were managed by consensus. Data extraction was performed by a team of two independent investigators (M.R. and D.M.) for each included study.

For each study, the following information was extracted: publication characteristics (authors, publication year and journal), study design (study type, clinical setting, number of patients/eyes included and duration of follow-up), baseline characteristics of the population (mean age, gender distribution, systemic and intraocular comorbidities, primary procedure, IOP, best corrected visual acuity (BCVA) and time since first intraocular surgery until the diagnosis of SCH), intervention (time from diagnosis of SCH until surgical management and short description of the surgical technique) and outcomes (final IOP and BCVA).

In the included studies, patients with spontaneous or traumatic SCH were excluded from our analysis, as well as patients who did not receive any surgical management of SCH.

The National Institutes of Health (NIH) Study Quality Assessment Tools were used to evaluate the risk of bias of the included case series and cohort studies [23]. For case reports, the CARE checklist was used [24].

## Results

### Literature search

**Fig. 1:**
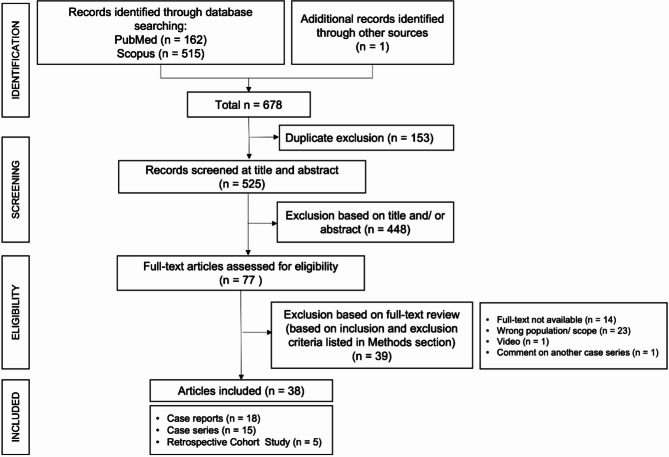
Summary of search and article selection according to PRISMA 2020 flow diagram for systematic reviews

A total of 678 publications were identified through our search of MEDLINE/PubMed (162 records) and Scopus (515 records) and one additional record was identified through other resources, namely citation searching. After removing the duplicates, 525 records remained. Following the title and abstract screening, we selected 77 articles for full-text review. After exclusion based on full-text review, we ended up with 38 articles for inclusion (Fig. 1). There were no sufficient data or number of high-quality longitudinal studies to endorse a meta-analysis. All the articles included were considered to have a quality rating of either good or fair.

## Studies characteristics

Table 2 summarizes the included studies. The studies were published between 2011 and 2022. Five retrospective cohorts were identified [5, 25–28], with most of the studies being case series (*n* = 15) [1, 4, 29–41] or case reports (*n* = 18) [2, 20, 42–57]. No randomized controlled trials were identified.

A total of 393 adults/eyes were included, varying from single case reports to the largest series of 112 patients/eyes [5]. The age of the patients ranged from 23 [38] to 93 years [37]. Excluding single case reports, the prevalence of men ranged from 33.3% [31] to 66.7% [40].

The primary surgery for the development of the SCH included cataract surgery (phacoemulsification [4, 30, 31, 39, 47, 54, 56] and ECCE [31, 41]), glaucoma surgery (trabeculectomy [2, 32, 38–40, 45, 48, 51], Ahmed valve implantation [38, 40], XEN Gel [42, 46] or PreserFlo Microshunt Implantation [44], nonpenetrating deep sclerectomy with HEMA implant [50], Baerveldt and Express tube [35, 37] and endocyclophotocoagulation [52]), pars plana vitrectomy (PPV) [1, 28, 31, 32, 40, 49, 53, 55, 57] and corneal surgery (PK) [32, 41].

SCH diagnosis was based on clinical features, including retinal fundus examination, confirmed in most of the reports with B-mode ocular ultrasound, which also assessed for its extent, exclusion of retinal detachment and to guide surgical drainage of the highest location of the hemorrhagic choroidal detachment.

The onset of SCH varied from being noticed intraoperatively (acute SCH (ASCH), mostly during complicated cataract extraction surgeries) to those diagnosed after 8 weeks (delayed SCH (DSCH), occurring more frequently after glaucoma surgery) [27].

The time from diagnosis of SCH until surgical management ranged from 0 [4, 44, 51] to 120 days [35]. The largest series were in accordance with a period until secondary surgical management between 7 and 14 days (range 1 to 32 days) [1, 5, 25–28, 31].

BCVA at diagnosis of SCH was generally poor: in most of the studies it varied between light perception (LP), hand motion (HM) and counting fingers (CF), with some patients presenting no LP [5, 29, 32]. Outcomes regarding the BCVA after the surgical management and at the end of the follow-up were variable, however, as depicted in Table 2, it is noteworthy that BCVA at last follow-up improved in the majority of the studies.

The few cohort studies in our search evaluated the visual outcomes in eyes with SCH according to its type (final BCVA of 1.2 logMAR (non-appositional SCH) vs. 1.86 logMAR (appositional SCH) (*p* = 0.002)) [5], its extent (final BCVA of logMAR 1.15 (one or two quadrants involved / limited SCH) vs. logMAR 2.60 (three to four quadrants of extent / full-blown SCH) (*p* = 0.002)) [26], timing of onset (final BCVA of 2.36 ± 1.67 logMAR (ASCH) vs. 1.92 ± 1.57 logMAR (DSCH) (*p* = 0.38)) [25] and type of surgical management (mean BCVA improvement of 1.18 ± 0.89 logMAR (drainage only group) vs. 0.32 ± 0.9 logMAR (combined PPV + drainage group) (*p* = 0.047)) [27].

**Table 2:** Summary table of the included studies

Author (Year)	Study Type	*N* OF EYES	Age (years)	Sex (Male, %)	Primary procedure	ASCH vs. DSCH	Timing of SCH (days)	Time since diagnosis until surgery (days)	Surgical procedure for management of SCH	BCVA before treatment of SCH	IOP before treatment (mmHg)	Follow-up	BCVA at last follow-up	IOP at last follow-up
Fan et al. (2022) [5]	Retrospective Cohort	112	79.9	54	Cataract, corneal or glaucoma surgery	DSCH (100%) (76 non-appositional SCH vs. 36 appositional SCH)	NR	10 to 14	Non-appositional vs. appositional SCH: observation in 46 (61%) vs. 12 (33%), delayed drainage in 14 (18%) vs. 15 (42%), delayed PPV in 16 (21%) vs. 13 (36%). A significant number of patients with appositional SCH were treated with combined delayed drainage and PPV (*n* = 7)	HM (logMAR 2.15, (all)); 2.03 logMAR (non-appositional SCH) vs. 2.39 logMAR (appositional SCH)	19.2 (SD 14.9)	3 months	1.2 logMAR (non-appositional SCH) vs. 1.86 logMAR (appositional SCH) (*p* = 0.002)	NR
Wang et al. (2022) [42]	Case report	1	86	100	XEN Gel Implant with mitomycin-C	DSCH	3	4	External drainage through a single sclerotomy in the superotemporal quadrant 8 mm posterior to the limbus	HM	5	2 months	5/10	10
Jiang H et al. (2022) [29]	Case series	5	70	60	Cataract surgery (80%); Glaucoma surgery (20%)	ASCH (80%)	0 to 1	10 to 14	Incision closure and AC deepening in the first surgery. Second drainage surgery with combined radial sclerotomy with 1–2 mm length (4–10 mm from the limbus) and PPV + PFCL + silicone oil or viscoelastic	LP or NLP	NR	6 months	3/10 (*n* = 1), LP (*n* = 1), NLP (*n* = 3)	NR
Lee T et al. (2022) [25]	Retrospective Cohort	50	73.5 ± 13.4	60	Cataract surgery (10%), Glaucoma surgery (72%), Vitreoretinal surgery (14%)	ASCH (30%)	14 ± 17 (min. 0)	23 ± 21	External drainage (45% underwent surgical drainage of SCH)	logMAR 0.67 ± 0.79 (all, *n* = 50) ; logMAR 1.41 ± 1.02 (ASCH) vs. logMAR 0.34 ± 0.36 (DSCH) (*p* = 0.003)	NR	At least 1 month	logMAR 2.05 ± 1.60 (all, *n* = 50) ; logMAR 2.36 ± 1.67 (ASCH) vs. logMAR 1.92 ± 1.57 (DSCH) (*p* = 0.38)	NR
Shekhar M et al. (2022) [26]	Retrospective Cohort	52	66.17 ± 9.71 (range 31–87)	36.5	Cataract surgery (100%)	ASCH (100%): limited (*n* = 28) and full-blown (*n* = 24)	0	7 to 14 (for full-blown ASCH)	External drainage + PPV + tamponade with silicone oil or PFCL	All: logMAR 2.45 (FCF); Limited SCH: logMAR 1.63 (6/240); Full-blown SCH: logMAR 2.60 (HM) (*p* = 0.004)	NR	3 months	All: logMAR 2.45 (FCF); Limited SCH: logMAR 1.15 (4/60); Full-blown SCH: logMAR 2.60 (HM) (*p* = 0.002)	NR
Koksaldi S et al. (2022) [20]	Case report	1	51	100	Complicated ECCE with anterior vitrectomy	ASCH	0	21	Immediate incision closure + IOP-lowering drugs + reverse Trendelenburg position in 1st surgery. Transscleral SCH evacuation with limited PPV in 2nd surgery	LP	NR	3 months	0.6 with aphakic contact lens correction	NR
Yeung et al. (2021) [2]	Case report	1	88	100	Trabeculectomy	DSCH - appositional	NR	NR	Three step technique: (1) AC maintainer + nonvalved 23- or 25-G trocar cannula at the highest peak of the SCH, 6–8 mm from and angled 20–30º toward the limbus. (2) Removal of the trocar to expose the sclerotomy ± insertion of a second trocar. (3) Focal peritomy around the preexisting sclerotomy and enlargement into a radial sclerotomy	CF	6	6 weeks	20/30	NR
Karaca U et al. (2021) [30]	Case series	5	74.6 (range 58–88)	60	Phacoemulsification	ASCH	0	11.4 (range 9–15)	Active external SCH drainage procedure with butterfly needle + 25G PPV + silicone oil or gas (C3F8 or SF6)	LP, HM or CF	10.4 (07–13)	At least 6 months	20/50 to 20/400	16.4 (14–18)
Boral SK et al. (2021) [31]	Case series	15	64.93 ± 7.62 (range 54–78)	33.3	Phacoemulsification (*n* = 10), ECCE (*n* = 1) and PPV (*n* = 4)	DSCH	NR	15.6 (range 6–32)	Transconjunctival 23G or 25G cannulaguided modified posterior passive drainage; 2–4 multiple sutureless posterior sclerotomies at 10–15 mm behind the limbus, vitrectomy with PFCL injection + silicone oil	logMAR 2.82 ± 0.21	27.87 ± 8.67	6 months	logMAR1.04 ± 0.53 (*P* < 0.001)	10.2 ± 5.16mmHg (*p* < 0.001)
Mo B et al. (2021) [1]	Case series	28	53.51 ± 10.21	57.1	PPV	ASCH (85.7%)	0	4	5 eyes were simply closely observed, 4 were given single SC drainage, 15 were given SC drainage combined with silicone tamponade, 2 underwent AC puncture, and 2 gave up treatment; 1 patient underwent a single SC drainage and was injected with r-tPA in the SC space on the fourth day after the occurrence of DSCH and drained after 4 h	NR	NR	24.94 ± 14.60 days	NLP to 20/30	NR
Akram H et al. (2021) [43]	Case report	1	85	0	Cataract surgery	NR	NR	4	Two-step approach: r-tPA (100 µg/0.1mL) in the superotemporal quadrant, 5 mm from limbus, 3 h before external drainage (two posterior sclerotomies 8 mm behind limbus) + 25-gauge PPV	LP	4	6 weeks	6/120 (aphakic)	NR
Qureshi A et al. (2021) [27]	Retrospective Cohort	17	70	50	Phacoemulsification (*n* = 7), corneal surgery (*n* = 5), glaucoma surgery (*n* = 3), vitreoretinal surgery (*n* = 2)	ASCH (40%)	0 days to 8 weeks	11 (range 1–22)	External drainage alone (full-thickness 2–3 mm scleral incisions) (*n* = 11) Vs. Combined drainage + 20 or 23G PPV + silicone oil (*n* = 9)	2.22 ± 0.26 logMAR (all); 2.16 ± 0.16 logMAR (drainage only group); 2.28 ± 0.35 logMAR (combined PPV + drainage group)	NR	19 (3–48) months	1.42 ± 1.02 logMAR (all); 0.98 ± 0.94 logMAR (drainage only group); 1.96 ± 0.89 logMAR (combined PPV + drainage group)	13.5 ± 5.3 (all); 14.3 ± 3.8 (drainage only group); 12 ± 7 (combined PPV + drainage group)
Micheletti E et al. (2020) [44]	Case report	1	76	100	PreserFlo Microshunt Implantation	DSCH	12	0 (first drainage) and 3 (second drainage + PPV)	2 stage surgery: sclerotomies isolated at first, then second surgery with repeated procedure + PPV + silicone oil endotamponade	LP	50	2 months	20/63	22
Rizzo S et al. (2019) [32]	Case series	6	66.7 (range 51–84)	50	PPV (*n* = 1), Silicone removal (*n* = 1) Trabeculectomy (*n* = 2), PK (*n* = 2)	DSCH (100%) (massive SCH with RD)	NR	12.3 (range 10–15)	2 stage surgery: first - drainage with 25G trocar + 25G PPV + PFCL; second - PFCL removal (mean permanency time of 11.8 days) and silicone oil introduction	LP (one patient without LP)	29.5	11 months (9–14)	LP to 20/200	10.8
Mears KA et al. (2019) [33]	Case series	4	NR	NR	NR	- (appositional)	NR	NR	Limbus-based anterior infusion line + drainage with a 25G trocar–cannula 8 mm posterior to the limbus + 25G PPV	NR	NR	NR	NR	NR
Středová et al. (2019) [45]	Case report	1	80	100	Trabeculectomy + 5-FU	DSCH -appositional	5	NR	23G 4 mm from the limbus + active drainage	LP	Hypotonic	1 year	6 / 30	NR
Stringa F et al. (2019) [46]	Case report	1	78	100	Anterior segment surgery with XEN	DSCH	7	7	Transscleral drainage	0.1	14	8 weeks	6 /12	18
Chai F et al. (2018) [34]	Case series	3	63 (range 56–73)	0	Cataract surgery	DSCH	5 to 10	NR	Sub-Tenon’s injection of urokinase one day before drainage with 20G incision in the SC space 3.5 mm from the limbus + PPV + silicone oil	LP (*n* = 1) and HM (*n* = 2)	8.5 (6.7–9.6)	9 months (6–14 months)	NLP or 20/1000	NR
Fei P et al. (2018) [47]	Case report	1	73	100	Phacoemulsification	ASCH (massive)	4	5 days until injection of r- tPA; 6 days until PPV	r-tPA (10 µg/0.2 mL) 1 day before injected into each quadrant of the SC space + external drainage (sclerotomy 5 mm behind the limbus in all 4 quadrants) + 25G PPV + lens fragment removal + C3F8 tamponade	LP	NR	10 months	30/60 after secondary IOL implantation	NR
Hussain et al. (2018) [48]	Case report	1	38	0	Trabeculectomy	DSCH (massive)	1	14	Posterior sclerotomy + 23G PPV + SF6	LP	NR	12 months	4/10	18
Sukpen I et al. (2018) [49]	Case report	1	32	0	PPV	ASCH	NR	NR	The air infusion pressure was elevated to 100 mmHg for several minutes to stabilize the SCH. No posterior drainage sclerotomy was created. Injection of C3F8 14%	20/400	NR	14 months	HM	NR
Rebolleda G et al. (2018) [50]	Case report	1	79	0	Nonpenetrating deep sclerectomy with HEMA implant	DSCH (extensive appositional)	1	7	Drainage + PPV	HM	18	1 year	HM	10
Kurup SK et al. (2017) [35]	Case series	6	76.7	50	Insertion of glaucoma seton devices (Baerveldt and Express tube)	DSCH (appositional)	7 to 14	36.5 (range 14–120)	Incisional sclerotomy 4 mm from the limbus + passive drainage + viscoelastic injection in the posterior segment (3 to 4.5mL) through the same sclerotomy. Additional partial-thickness sclerotomies were created in the other quadrants	LP (*n* = 4) and CF (*n* = 2)	NR	8 months	5 eyes with 20/30 to 20/200 (one patient with LP - refused surgery)	< 28 mmHg
Lin HZ et al. (2016) [51]	Case report	1	74	100	Trabeculectomy with mitomycin-C	DSCH	3	0	2 circumferential sclerotomies 4 mm from the limbus and 3 mm in length were performed in the superior nasal and superior temporal quadrants. The SCH was then drained from the 2 sclerotomies with constant irrigation pressure from the AC	LP	49	6 months	20/25	16
Al-Asbali et al. (2016) [52]	Case report	1	35	0	Endocyclophotocoagulation	ASCH (appositional)	0	7	Drainage through two temporal sclerotomies	LP	26	5 months	20/400	24
Savastano A et al. (2016) [4]	Case series	6	68.2 ± 4.5	NR	Phacoemulsification	ASCH	0	0	Stop the surgery + extreme reverse Trendelenburg position + rapid IV infusion of mannitol solution, IV midazolam and sublingual nifedipine. After 30 min to 1 h, conclusion of primary surgery + 10/0 nylon stich to the corneal tunnel	NR	NR	4 months	6/15 − 6/7.5	14–18
Laube T et al. (2015) [36]	Case series	3	83 (range 74–89)	0	Cataract surgery	DSCH (67.7%)	0 to 21	12.3 (range 5–23)	Transscleral drainage of SCH + PPV + PFCL + silicone oil tamponade	LP	NR	16.3 ± (5–26) months	20/20 (*n* = 1), 20/320 (*n* = 1), HM (*n* = 1)	NR
Reibaldi M et al. (2015) [28]	Retrospective Cohort	39	67 ± 6	61.5	PPV	DSCH	1 to 2	12 ± 5	21 eyes underwent combined drainage and vitrectomy simultaneously, 7 had drainage only.	LP to 20/80	34.5 ± 5.8	27 ± 8 months	1.6 logMAR (20/800)	Hypotony or phthisical
Vuković D et al. (2015) [53]	Case report	1	56	100	PPV	ASCH	0	21	Drainage through PPV ports + PFCL + silicone oil	LP	1	4 weeks	4/60	NR
Dreyer EB et al. (2014) [37]	Case series	2	45 and 93	50	Implantation of Ex-Press miniature glaucoma device + intraocular lens	DSCH (100%)	1	14	Drainage only through 2 sclerotomies 7 mm behind the limbus	LP	17 and 23	NR	20/200 and LP	NR
Pakravan M et al. (2014) [38]	Case series	5	55.2 (range 23–81)	60	Trabeculectomy (*n* = 4), Ahmed valve implantation (*n* = 1)	DSCH (100%)	NR	within 36 h	Early Choroidal Tap + AC Reformation: 2 circumferential scleral incisions 4 mm in length, 4 mm from the limbus, in the inferior quadrants. Milking massage for drainage. BSS in the AC during choroidal tap.	LP (*n* = 3) or HM (*n* = 2)	37 (25–45)	17.2 (range 6–40) months	20/120 (*n* = 1), 20/200 (*n* = 2), CF (*n* = 2)	17.2 (15–20)
Jin W et al. (2014) [39]	Case series	6	61.7 (range 40–75)	50	Phacoemulsification (*n* = 3), trabeculectomy + mitomycin-C (*n* = 2), silicone oil removal (*n* = 1)	DSCH (100%)	NR	2.2 (range 1–5)	Drainage + PPV + silicone oil (*n* = 3), only drainage (*n* = 3)	LP (*n* = 4), HM (*n* = 2)	43.3 (30.1–56.7)	15 months (12–18)	HM (*n* = 3), CF (*n* = 1), 0.4 (*n* = 2)	18.8 (16.1–21.3)
Lavinsky F et al. (2013) [40]	Case series	9	74 (range 61–84)	66.7	Glaucoma surgery (*n* = 4), cataract surgery (*n* = 3), PPV (*n* = 2)	ASCH (33%)	0 (*n* = 3), 6 ± 3 (range 1–8) (*n* = 6)	11 ± 4 (*n* = 6)	Posterior drainage sclerotomies (5–8 mm posterior to the limbus and extending 3–5 mm posteriorly) only (*n* = 3) or with PPV (*n* = 6) + silicone oil (*n* = 5)	HM	NR	38.3 (4–87) months	20/385	NR
Valldeperas X et al. (2012) [54]	Case report	1	69	0	Phacoemulsification	ASCH (massive)	0	1	3 radial drainage sclerotomies + PPV + PFCL + heavy silicone oil + supine positioning	HM	43	2 months	3/60	NR
Wagley S et al. (2012) [55]	Case report	1	61	0	PPV	DSCH	6	18	3 radial 5 mm drainage sclerotomies, 4 mm behind the limbus	LP	NR	3 months	20/40	NR
Ghorayeb G et al. (2012) [56]	Case report	1	61	100	Phacoemulsification	DSCH	3	16	External drainage through 2 sclerotomies 2 mm posterior to the limbus	CF	6	11 days	20/20	14
Rezende FA et al. (2012) [41]	Case series	2	71 and 82	50	ECCE, PK	ASCH (100%)	0	14	A 20G transconjunctival trocar/cannula system was inserted into the SC space 7 mm from limbus for external drainage. Associated PPV in 1 patient	LP	NR	6 months	20/80 and 20/400	14 and 15
Kunjukunju N et al. (2011) [57]	Case report	1	62	100	PPV	ASCH	12	1	r-tPA 100 µg into the AC and SC space 15 min before surgery / 45 min before drainage + external drainage with sclerotomies + PFCL + silicone oil	LP	43	8 months	20/40	NR

Legend: When sample size *n* > 2, the values corresponded to the mean value. Abbreviations: 5-FU: 5-fluorouracil, AC: anterior chamber, ASCH: acute suprachoroidal hemorrhage, BSS: balanced salt solution, CF: counting fingers, DSCH: delayed suprachoroidal hemorrhage, ECCE: extracapsular cataract extraction, FCF: finger counting close to face, HM: hand motion, IOP: intraocular pressure, IV: intravenous, LP: light perception, NLP: no light perception, NR: not reported, PFCL: perfluorocarbon liquid, PK: penetrating keratoplasty, PPV: pars plana vitrectomy, RD: retinal detachment, r-tPA: recombinant tissue plasminogen activator, SC: suprachoroidal, SCH: suprachoroidal hemorrhage, SD: standard deviation

## Discussion

Several questions concerning the management of SCH remain a matter of debate. We intended to gather all the surgical records of our literature search and summarize them, highlighting some novel variations, as outlined in Table 2.

### Indications for surgical drainage – who?

When the SCH is non-appositional, limited to one or two quadrants, medical treatment (including observation, cycloplegic agents, corticosteroids and IOP-lowering drugs) may be effective and it may not need surgical intervention [3, 58]. Fan et al. also reported SCH’s management only with observation when VA was no LP on presentation [5].

Surgical drainage might be indicated in cases with flat AC, uncontrolled IOP, refractory pain, retinal detachment, kissing choroidal (appositional SCH), extent of SCH in more than 2 quadrants (full-blown SCH), macular involvement and incarceration of intraocular content into the surgical wound [3, 21, 26, 58, 59].

### Optimal timing of intervention – when?

When indicated, the timing for surgical drainage of the SCH is also a topic which lacks agreement between different studies. Many authors report an optimal timing between 7 and 14 days after the diagnosis of SCH, which corresponds to the expected time for clot lysis, that can be monitored using serial B-scan ultrasound [2, 3, 20, 26]. Upon reviewing the cohort studies, we observe that the timing for surgical intervention for SCH generally occurred around the second week (10–14 days) after the diagnosis of SCH [5, 26–28].

Pakravan et al. introduced an alternative approach for treatment of DSCH, where instead of the usual 7 to 14 days period for clot liquefaction, the SCH was drained immediately after being diagnosed. This approach resulted in a significant decrease of IOP after the intervention (*p* = 0.01) and in a statistically significant improved visual outcome (final BCVA of 1.09 ± 0.31 logMAR (*p* = 0.003)). The authors argued that a considerable quantity of blood within the eye may worsen intraocular inflammation and increase the risk of intraocular adhesions, requiring a more complex intervention in the future and, possibly, implying worse outcomes, which can be avoided if drained immediately [38]. However, a more recent cohort study from Lee et al. [25] showed that surgical drainage of SCH did not significantly affect final visual outcome after controlling for pre-drainage VA (*p* = 0.06). Further clarification is needed to determine the functional impact of surgical drainage in cases of SCH.

The use of exogenous fibrinolytic agents to accelerate clot lysis and to allow an earlier drainage of SCH is promising. A study from Wagley et al. measured the concentration of tissue plasminogen activator (t-PA) in liquefied SCH, reporting higher levels (threefold) when comparing to the serum. Hence, t-PA may play an important role in SCH liquefaction [55]. Recombinant t-PA (alteplase) and urokinase-type plasminogen activator have already been tried and described in several case reports and case series [2, 34, 43, 47, 57]. The site of injection (alteplase in the suprachoroidal space [2, 43, 47, 57] 5 mm from the limbus [43] or urokinase in the sub-Tenon’s space [34]), extent (in the quadrant where the height of the SCH is greater [43] versus all four quadrants [47]), the timing (15–45 min [2, 57], 3 h [43] or 1 day before drainage [34, 47]), and dose (alteplase 10 µg/0.2 mL [47], 100 µg/0.1 mL [43, 57] or 12.5 to 50 µg [2]) varied, but, in general, its use was considered promising. Larger studies and randomized controlled trials should be highly encouraged in the future.

### Surgical approach of SCH – how?

Regarding the surgical approach of SCH, there are many controversies. The surgical management varies according to the time of onset of SCH. When occurring intraoperatively (mostly in cataract surgery), the intervention consists in prompt closure of the wound (with 10/0 nylon sutures, for example) and repositioning of the prolapsed tissues, when present and if possible [20, 45]. Immediate interruption of the surgical procedure is useful in limiting the evolution of an expulsive SCH during cataract surgery [4]. The reverse Trendelenburg position in primary procedure can also be beneficial because it decreases the central venous pressure and subsequently reduces the blood flow into the choroidal layer [4, 20]. Wound closure leads to an increase of the IOP, which serves as a tamponade of the hemorrhage, stabilizing it [45, 49]. The performance of sclerotomies in the acute phase of SCH is controversial. If the globe can be successfully sutured and left normotonic or slightly hypertonic, SCH drainage should be delayed, in order to reduce the risk of rebleeding [20]. Drainage in the primary surgery can be unsuccessful due to quick blood coagulation and the risk of becoming impossible to evacuate it. Therefore, performing sclerotomies in the acute phase is considered not appropriate for the eye [45, 60]. However, if the high IOP threatens the vitality of the optic nerve, drainage can be done intraoperatively through posterior sclerotomies [20]. Valldeperas X et al. reported a successful case of massive ASCH submitted to early drainage, 24 h after the primary surgery of phacoemulsification. The patient was submitted to three radial sclerotomies with combined PPV using perfluorocarbon liquid (PFCL) and heavy silicone oil (Densiron 68^®^) as an endotamponade, and the patient was instructed to stay in supine position for one week. His vision improved from HM to 3/60. The authors justified that this early intervention would protect the extension of the SCH posteriorly and also prevent other late complications such as retinal detachment and proliferative vitreoretinopathy [54].

Nevertheless, the precise timing for drainage is controversial, which also applies to postoperative/ delayed SCH (for further discussion of drainage of DSCH, see the topic “Optimal timing of intervention – When?”).

Any applicable subsequent surgical treatment is highly individual, which in part explains the limited number of cohort studies, in comparison to a higher number of case reports and case series with a small sample size.

Yeung et al. [2] detailed a complete stepwise surgical approach for drainage of SCH, which first involves the introduction of an AC maintainer (dispersive viscoelastic device or balanced salt solution (BSS)) to pressurize the globe and also to promote the passive outflow of the choroidal hemorrhage [58]. It also should be done in cases where the surgeon is not sure of the correct placement of the posterior infusion cannula in the vitreous cavity (when PPV is performed), since a wrong positioning of the cannula in the subretina can eventually lead to complications [2].

External drainage is achieved through one or more sclerotomies, after conjunctival peritomy for scleral cutdown (circumferential [38, 51] or radial [29, 54, 55]) or by performing transconjunctival sclerotomies using ports for PPV [31, 41]. Yeung et al. [2] described the sclerotomy with a nonvalved 23- or 25-gauge trocar inserted 6–8 mm from the limbus (in the literature the distance varied between 2 mm [56] and 15 mm [31]), oriented at 20 to 30 degrees (or 5 to 10 degrees tangential to the scleral plane [31]) towards the limbus to access the suprachoroidal space. The site for drainage should be at the greatest point of the SCH, assessed by B-mode ocular ultrasound [2]. Afterwards, the surgeon ought to enhance the infusion pressure in the AC or in the vitreous cavity (to 35mmHg [30], 45mmHg [32], 40-60mmHg [31] or even 60-100mmHg [49]) and should applicate external pressure by using a cotton tipped applicator [2]. If the drainage of SCH is still incomplete, a 26-gauge guarded needle attached to active aspiration can be inserted, as well as another 23- or 25-gauge trocar in a distinct quadrant (using the same technique described above). If after these steps the drainage is still inadequate, the trocar cannulas inserted in the suprachoroidal space can be removed in order to expose the sclerotomy and, as a last step, the sclerotomy can be expanded into a 2–3 mm radial sclerotomy, which should finally lead to spontaneous drainage of SCH [2]. These authors report that this drainage technique is less invasive than the typical scleral cutdown procedure, which involves a considerable conjunctival cutdown, rectus muscle manipulation and increased risk of hypotony, perforation or retinal injury [2, 58].

Drainage of the hemorrhage is usually achieved passively, but alternatively, it is possible to perform active aspiration. In the series of Karaca et al., the standard three-port 25-G trocar system was used to perform sclerotomies, the infusion pump of the system was turned on and IOP was set to 35 mmHg. The choroidal hemorrhage was passively drained initially through a standard sclerotomy 8 mm from the limbus, then a butterfly needle was placed into the vitrectomy suite’s aspiration port and the program was set to extrusion mode. The suprachoroidal space was entered with the butterfly needle on bevel-up position and the SCH was subsequently aspirated. This novel technique managed to achieve continuous drainage of SCH in a significant amount, which consequently led to a faster recovery time and better visual outcomes [30].

During PPV, additional passive drainage is possible with instillation of PFCL as an adjunct to fill at least half of the vitreous cavity and to press the SCH uniformly from the inside [26, 29, 31, 32, 36, 53, 54, 57]. PFCL is then removed, PPV is continued and at the end of the surgery, silicone oil (more frequently), gas (C3F8 [30, 47, 49] or SF6 [30, 48]) or BSS is injected as tamponade after air–fluid and air–PFCL exchange [30]. PFCL is frequently removed intraoperatively, but Rizzo et al. described a novel technique in eyes with massive SCH, leaving PFCL in the vitreous cavity (full volume) during a fortnight. In order to enhance the tamponade action of PFCL, which itself has a higher tamponade effect than silicone oil, the patients were positioned face-up, to help squeezing the remaining SCH in the days after the surgery. After 10 to 14 days (mean PFCL permanency time of 11.8 days), the patients underwent a second surgery for PFCL replacement by silicone oil. None of the patients were found to have residual choroidal detachment and, in all cases, the retina seemed to be flat, with no evidence of epiretinal or subretinal proliferative vitreoretinopathy [32].

Likewise, other substances have been reported to be left in the vitreous cavity, namely a dispersive viscoelastic agent (Viscoat^®^). Kurup et al. published a series of six eyes with DSCH submitted to pars plana choroidal drainage along with simultaneous off-label application of viscoelastic (3 to 4.5mL) into the posterior segment (in patients with concomitant posterior vitreous detachment), which was only approved for use in intraocular surgeries of the anterior segment [35].

Although the majority of studies regarding the surgical approach of SCH describe procedures comprehending PPV, Rezende et al. suggested that PPV might not be necessary in all cases, stating that it should only be done when there is concomitantly a condition that requires vitrectomy (such as vitreous hemorrhage and retinal detachment or incarceration) [41].

### Visual prognosis (BCVA) – does the surgery improve vision?

As mentioned in the results section, there is a very limited number of retrospective cohort studies and large case series in the literature. Those we found in our research evaluated the visual outcomes in eyes with SCH according to its type [5], extent [26], onset of SCH (ASCH vs. DSCH) [25] and surgical management (drainage vs. no drainage [25] and drainage only vs. combined drainage and PPV [27]).

Fan et al. [5] demonstrated that appositional DSCH (*p* = 0.01) and duration of apposition (*p* = 0.04) were correlated with worse visual outcomes and that observation remains a reasonable management strategy for non-appositional SCH. Similarly, Shekhar et al. studied the functional outcomes of ASCH according to its extent, with eyes with limited SCH having better visual outcomes in comparison to eyes with full-blown SCH [26].

Regarding the decision for drainage (irrespective of ASCH or DSCH), Lee et al. reported that surgical drainage of SCH did not have an impact in final BCVA, after controlling for pre-drainage VA, in comparison with SCH that were not drained [25]. In contrast, Qureshi et al. found that in eyes with extensive SCH, surgical drainage with or without PPV did in fact improve final BCVA [27]. No statistically significant differences between both groups were found concerning the proportion of patients who achieved functional success (defined as BCVA improvement of 2 or more logMAR lines when compared to baseline), recommending combined drainage and PPV with silicone oil tamponade only in more complex cases of SCH with vitreoretinal involvement [27]. Nevertheless, these authors did not compare these groups (drainage only and combined drainage with PPV) with a third group not receiving any surgical intervention, as opposed by Lee et al. [25]. Therefore, further studies are needed to assess the real impact of surgical drainage on VA in patients with SCH.

### Limitations

As previously mentioned, the lack of studies with higher levels of evidence such as randomized controlled trials, cohort or case-control studies constitutes the major limitation of this review. The rarity of this surgical complication and the myriad of surgical techniques, many times at surgeons’ discretion, justifies the need for including case reports and small case series in this systematic review. However, data extraction and compilation of clinical variables, when present, were challenging, due to the heterogeneity of the reports. Furthermore, some studies were included for reporting that surgical drainage was performed, however they did not describe the surgical technique itself.

## Conclusion

To the best of our knowledge, this is the first systematic review of the surgical management of perioperative SCH. It is a very serious and feared complication of intraocular surgeries, although fortunately extremely uncommon. Based in our review, we acknowledge that the optimal timing for intervention still remains unclear, although it points towards the second week after SCH installation. Since there is no standardized surgical approach of SCH, further clinical research is on demand to improve the management of this clinical sight-threatening entity.

## Data Availability

No datasets were generated or analysed during the current study.
